# Thermophilic versus Mesophilic Anaerobic Digestion of Sewage Sludge: A Comparative Review

**DOI:** 10.3390/bioengineering3020015

**Published:** 2016-06-18

**Authors:** Getachew D. Gebreeyessus, Pavel Jenicek

**Affiliations:** 1 Urban Environmental Management, Kotebe University College, 31248, Addis Ababa, Ethiopia; 2 Water Technology and Environmental Engineering, University of Chemistry and Technology, Technická 5, 166 28 Prague 6—Dejvice, Czech Republic; Pavel.Jenicek@vscht.cz

**Keywords:** dewaterability, mesophilic anaerobic digestion, reject water, rheology, sludge, thermophilic anaerobic digestion

## Abstract

During advanced biological wastewater treatment, a huge amount of sludge is produced as a by-product of the treatment process. Hence, reuse and recovery of resources and energy from the sludge is a big technological challenge. The processing of sludge produced by Wastewater Treatment Plants (WWTPs) is massive, which takes up a big part of the overall operational costs. In this regard, anaerobic digestion (AD) of sewage sludge continues to be an attractive option to produce biogas that could contribute to the wastewater management cost reduction and foster the sustainability of those WWTPs. At the same time, AD reduces sludge amounts and that again contributes to the reduction of the sludge disposal costs. However, sludge volume minimization remains, a challenge thus improvement of dewatering efficiency is an inevitable part of WWTP operation. As a result, AD parameters could have significant impact on sludge properties. One of the most important operational parameters influencing the AD process is temperature. Consequently, the thermophilic and the mesophilic modes of sludge AD are compared for their pros and cons by many researchers. However, most comparisons are more focused on biogas yield, process speed and stability. Regarding the biogas yield, thermophilic sludge AD is preferred over the mesophilic one because of its faster biochemical reaction rate. Equally important but not studied sufficiently until now was the influence of temperature on the digestate quality, which is expressed mainly by the sludge dewateringability, and the reject water quality (chemical oxygen demand, ammonia nitrogen, and pH). In the field of comparison of thermophilic and mesophilic digestion process, few and often inconclusive research, unfortunately, has been published so far. Hence, recommendations for optimized technologies have not yet been done. The review presented provides a comparison of existing sludge AD technologies and the gaps that need to be filled so as to optimize the connection between the two systems. In addition, many other relevant AD process parameters, including sludge rheology, which need to be addressed, are also reviewed and presented.

## 1. Introduction

WWTPs are huge liquid waste management facilities for the existing and emerging cities worldwide. As a by-product of the WWTPs, a huge amount of primary and secondary sludge is being produced there and managing it is still a big challenge. For instance, in the European Union (EU) alone, about 30,000 tons of sludge dry mass are generated every day with an extra possible projection of a 10% increase by 2020 [1]. Economically, up to 30% of the capital cost and 50% of the operational cost of WWTPs is taken up by the sludge management work [2]. The economic inefficiencies of WWTPs and the huge volume of sludge with regard to its transport and management as well as stabilization costs are still challenges that have to be dealt with by professionals in the field in order to optimize cost of these WWTPs. One approach to these challenges is an exploitation of the recoverable resources contained in sludge.

Though landfilling and incineration are common practices for sludge management, recently their environmental and public opinion related impacts have triggered the need for better management alternatives. As a result, the reuse of sludge for land application and recovery of energy is getting more attention. In this regard, the anaerobic digestion (AD) of sewage sludge is favored for its advantages over other sludge management alternatives. These include recovery of clean energy (biogas), a reduction of 30% to 50% of sludge amounts, destruction of pathogens, removal of bad odor, and some others [3].

The transport of sludge, the digester mixing energy requirement, digester heating energy, and energy for pumping sludge are the main issues in most cases. Hence, dewatering the water in the sludge is a necessary task in the WWTPs. 

The water in the sludge could exist in different forms; free, capillary, vicinal and chemically bound with the solid that is in suspension. Thus, the dewatering technology that could be preferred has to be able to remove those water forms in the sludge. Moreover, the water in the sludge has to be considered as a recoverable resource, as about 99% of the raw sludge is water by content [4]. Several researchers have reported that the dewatering ability of mesophilic and thermophilic AD sludge is different. In relation to this, the temperature of sludge AD has to be optimized regarding to sludge dewaterability. Other important parameters and influenced by temperature are: the biogas yield, reject water ammonia content, as well as AD foaming phenomena.

Concerning energy advantages, the thermophilic sludge AD is preferred to the mesophilic sludge AD [5,6,7]. Concurrently, issues of process stability in relation to operational conditions are dealt with, comparing these two thermal systems [8,9], in favor of the mesophilic. On the other hand, some studies claim that it is not any problem with the thermophilic AD if adaptation of biomass is long enough. Therefore, these AD process stability related studies have to be normalized for possible comparisons. Furthermore, there are conflicting issues still on the biogas yield and the reject water quality as well as the digestate quality [10,11,12] between the sludges of mesophilic and thermophilic ADs. 

Thus, it is hypothesized in this review paper that temperature of the AD would have a consequence on the dewaterability of the sludge, the reject water quality and the foaming phenomena. Therefore, reviewing articles and comparing mesophilic and thermophilic sludge AD is important to identify such related gaps, in order to achieve process optimization. Afterwards, this review suggest the ways for optimization of sludge AD among biogas yield, dewater ability, and reject water quality, as it is an important issue in the forthcoming research.

## 2. Typical Source and Composition of Sewage Sludge

### 2.1. Massive Sludge Sources

**Figure 1:**
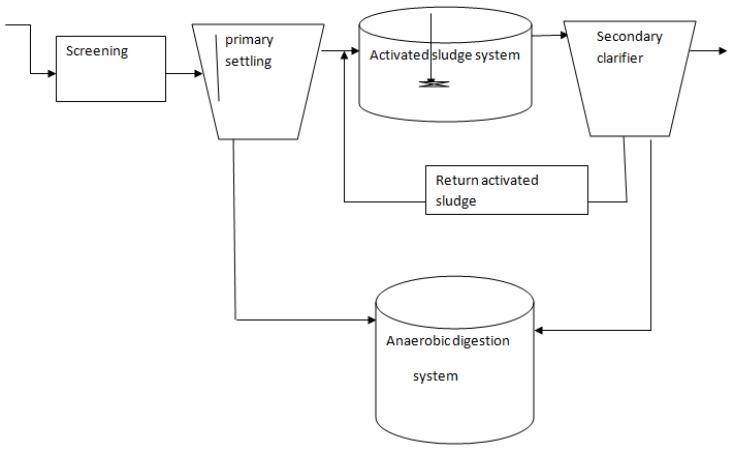
Major processes in the conventional wastewater treatment system.

Conventionally, wastewater sludge is a by-product of the primary and secondary settling operations at the WWTPs. In that case, the conventional wastewater treatment process involves mainly three unit operations following screening and related preliminary treatments. The first of which is primary settling that is performed with the possible addition of coagulant chemicals to enhance the settling of the particles in the wastewater. By dosing coagulants, much of the solids are settled out, mainly the organic masses that are unstable, and such residues are useful inputs to AD plants (Figure 1). Therefore, the first unit operation to give massive sludge at the WWTPs is the primary settling.

Consequently, the supernatant of the primary clarifier goes in to the activated sludge system unit where consortia of organisms, mainly bacteria and protozoa, degrade the organic substrates in to still nutrient rich effluent and biomass rich sludge known as the waste activated sludge (WAS). Obviously, there the strength of the waste and hence the extent of stabilization is evaluated and is expressed as the chemical oxygen demand (COD) of the wastewater. COD is thus an indirect expression for the strength of waste that is based on the amount of oxygen required to oxidize the organics.

The WAS is then settled in a secondary clarifier. The secondary sludge settled in the secondary clarifier is partly recycled into the activated sludge process unit and partly goes to the sludge digestion unit. The supernatant that is still rich in nutrients and probably contains pathogens is followed by disinfection and nutrient removal based advanced processes. Those polishing unit processes help to comply with discharge limits. 

### 2.2. Sludge Composition and Discharge Limits

Generally, composition of sewage sludge is a function of the parent material and the subsequent treatments applied to it. The core compositions of typical sludge are organic compounds expressed in terms of the volatile solid (VS) or COD as stated earlier, suspended solids (SS) or dissolved solids (DS), and nitrogen and phosphorus. 

The sludge composition is classified into the following categories according to the report of the European Commission, 2001.

A: primary sludge, primary sludge with physical/chemical treatment or high pollution load;
B1: biological sludge (low load);
B2: biological sludge from clarified water (low and middle load);
C: mixed sludge (mix of A and B2 types);
D: digested sludge. The respective sludge composition is thus depicted in Table 1.

In relation to wastewater sludge, various national and international rules are set to be met before discharge of the supernatant and the sludge into the natural environment from those conventional WWTPs. For example, the EU has regulation under the title of Urban Wastewater Treatment Directive 91/271/EEC, which sets the minimum levels prior to discharge to surface water that is assigned based on the population size. Especially, the nitrogen and phosphorus limits apply to sensitive areas, mainly to those of natural freshwater lakes. Similarly, there are rules with regard to pathogens, odours and stability of sludge. Thus, in an effort to optimize AD systems, the reject water quality needs to be considered from the point of view of meeting discharge limits.

## 3. Sludge Anaerobic Digestion and Process Stability

Process stability, the long start up period and the needed process time, mal-odorous nuisances mainly due to H_2_S, and vulnerability to xenobiotic compounds are the most common process stability concerns with regard to AD in general where sludge AD is not exceptional. In that respect, optimization of AD temperature is subject to process stability as well.

**Figure 2:**
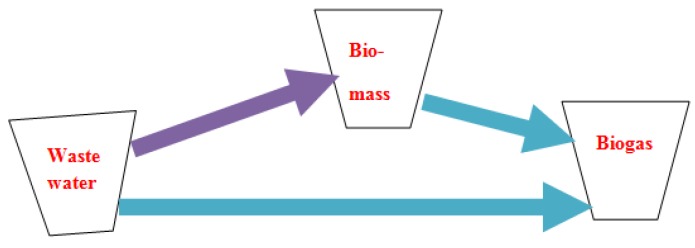
The possible route to biogas production during biological wastewater treatment.

Process stability in AD is also partly a function of the type of substrate involved in the AD. Thus, the higher the degradability of the substrate used in the AD, the bigger the issue of process instability. With respect to sludge AD, biogas can be recovered from the raw sewage sludge directly, which is mostly difficult to manage, or from use of the WAS or a mixture of the two (Figure 2).

**Figure 3:**
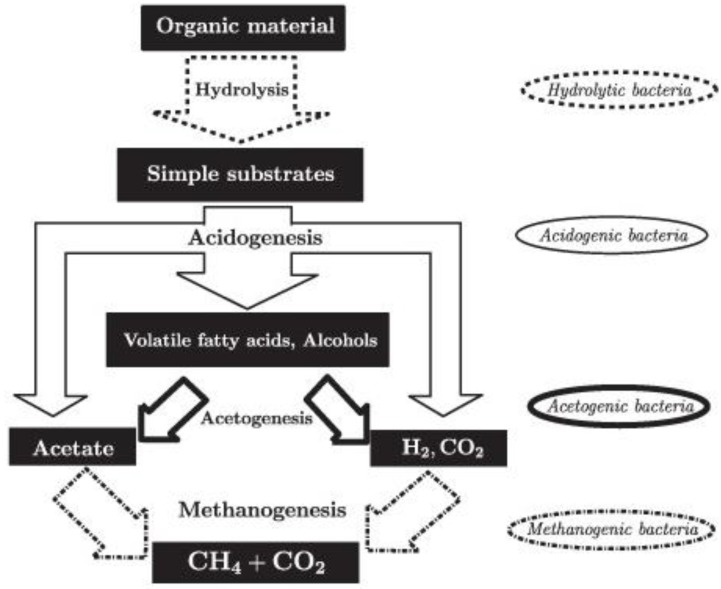
Schematic view of the various stages of anaerobic digestion processes. Source: [16].

Process stability in AD is attributed to different stages of degradation by the various organisms involved that may impact the thermophilic process differently from that of the mesophilic. As a system, AD consists of a series of microbiological processes that convert organic compounds to methane and carbon dioxide as well as hydrogen sulfide and water in the absence of oxygen. It reduces the VS by 35%–60%, depending on the operating conditions. As a complex and syntrophic microbiological process, AD mainly involves bacteria and archaea. The major syntrophic processes can be classified in to four stages: hydrolysis, acidogenesis, acetogenesis and methanogenesis, and these processes happen at the same time with different percentages of conversions (Figure 3). In most cases, the 70% methanogenesis is from the acetate metabolism, and the remaining 30% is from the hydrogenotrophs. 

In most cases, the hydrolysis stage is process rate limiting, which splits polymers mainly into monomers and oligomers. The acidogenesis stage process is about the transformation of monomers into short and medium chain acids and alcohols. This stage is a concern due to the acidification of digesters that marks the need to monitor digesters to avoid process failures or stuck. Furthermore, the acetogenesis stage is the conversion of short and medium chain acids to hydrogen, carbon dioxide and acetate. Lastly, in the methanogenesis stage, the carbon dioxides, hydrogen and acetate are converted to methane and water. 

The conversion of acetate to methane is the major pathway in thermophilic AD. All of the processes take place under very low oxidation reduction potential with reference to hydrogen, and, hence, for the methanogenesis, the reduction potential goes down to −300 mV or it has to be less than −200 mV [2]. The methanogenesis stage is performed by the most sensitive microbes of the anaerobic process that belong to the third domain of life in taxonomy—the archaea. The two major species within archaea are the hydrogenotrophic ones, which feed on hydrogen, and the acetoclastic ones, which feed on the acetate intermediate. The common species involved here are *Methanosaetaconcelii*, *Methanobacteriumbeijingense* and *Methanosarcinaformcoccus*, to mention a few [14,15]. 

These degradation processes are prone to be upset. Although the overall process in AD is exergonic (the change in Gibb’s free energy is negative), some steps such as propionate oxidation are sensitive to build-up of H_2_, which is an issue of concern with process stability. This process has to be in syntrophy with methanogens or sulfur reducers to proceed under standard conditions. Kaspar and Wuharmann clearly stated that hydrogen and acetate are inhibitors of microbial degradation of propionate and ethanol, hence the partial pressure of hydrogen and alkalinity have to be used as a monitoring parameters to sludge AD [17,18]. 

In complex waste mixtures like the sewage sludge, 30% of the electron flow occurs through oxidation of fatty acids. Thus, there is high production of the diatomic hydrogen, which has to be reduced by the responsible organisms in order for the process to proceed safely. 

Propionate is the most produced intermediate in AD of organics. Propionate oxidation produces hydrogen molecule whose partial pressure has to be between 10^−4^ and 10^−6^ atmosphere for the system to operate efficiently, while for the oxidation of ethanol, its value should range between 10^−1^ and 10^−6^ [8]. 

Appels and colleagues noted that process stability and control is more an issue for the thermophilic anaerobic reactors compared to the mesophilic ones. The reason for that is due to the increase of free ammonia in the system that inhibits the microorganisms therein. The amount of free ammonia in AD systems depends on the total ammonia, temperature and pH in the system. Free ammonia increases with pH and temperature. The resulting instability leads to an increase of volatile fatty acids (VFAs) that causes a decrease in pH and hence a decrease in free ammonia that the process remains to a certain extent self-regulating. However, the amount of methane production is reduced based on the ammonia level. 

In such a case, a free ammonia concentration in the range of 560–568 mg NH_3_-N/L leads to inhibition of 50% of the methanogenesis at pH 7.6 under thermophilic conditions, and a total AD process inhibition can occur at a concentration of 10 g NH_3_-N/L. A VFA concentration of 2 g/L is also reported to inhibit cellulolytic hydrolysis, alarming that its concentration as acetic acid has to be kept lower than the inhibitory concentration level [2,19,20]. Generally, ammonia concentration of less than 200 mg/L is desired for the AD, as nitrogen is also necessary for microbes if it is in a desired range of concentration (Table 2). 

Regarding the VFAs, the acids are diverse in ADs and have a joint effect on the bacteria and archaea working therein. Thus, the individual concentrations of those short as well as medium molecular sized acids do matter in the AD process stability with varying magnitude in impact. For example, an acetic acid concentration of 2400 mg/L and butyric concentration of 1800 mg/L do not have an inhibitive effect on methanogens. However, a propionic acid concentration of 900 mg/L resulted in a significant inhibition of the methanogens [21]. This emphasizes the need to monitor the level of propionate acids in ADs. 

Rubia et al. stated in their review article that another issue of process stability of concern with the thermophilic digestion of sludge is related to the procedure in which the thermophilic inoculum is taken. As a result, the reviewers recommended that a fast increase in temperature to the target level with the gradual raising of the organic loading rate (OLR) can be an approach to this problem. 

Hence, the choice of an AD technology is linked to process stability. In a related fact, a limitation with a single-stage continuously stirred tank reactor (CSTR) process configuration technology is due to its little stability. This is why CSTRs are taking longer amounts of time to reach process equilibrium, or they could even fail to reach equilibrium as compared to the two-staged configuration [22]. 

## 4. Thermophilic versus Mesophilic Sludge Anaerobic Digestion

### 4.1. Comparison of Mesophilic and Thermophilicanaerobic Digestion of Sludge

In most studies, comparison of mesophilic and thermophilic AD is geared towards the biochemical process speed and biogas yield. Some authors describe that the promising advantage of thermophilic sludge AD compared to mesophilic AD is better biogas and better digestate quality, though most findings are inconclusive. In addition, the kinetics of whole-sludge AD have been studied, and the results showed that the rate is faster in thermophilic AD than in mesophilic in general, which is ascribed to the temperature effect in speeding up the biochemical reaction rate [6,10]. 

Furthermore, the relative digestion kinetics of pure substances is investigated targeting the particular step that could be affected most by temperature variation during the AD process. In this regard, Ge et al. found out that the hydrolysis rate is the most temperature influenced stage that strongly followed the Arrhenius relationship, with activation energy of 31 ± 4 kJ·mol^−1^, and an increase of process speed 1.5 times for each 10 °C of temperature rise is recorded. In a related recent study on WAS, the methane production is significantly improved (from 35.8% to 48.2%) in the thermophilic case compared to its mesophilic counterpart. Furthermore, the authors, Li et al, found out that the rate limiting stages of anaerobic process affected by temperature includes the acidogenesis in the mesophilic case in addition to the hydrolysis phase [23]. 

Mesophilic AD has been widely in practice in most cases because of its lower process energy demand for reactor heating and its better stabilization. Conversely, thermophilic AD is preferred for its high biochemical reaction and low retention time in addition to stabilization. Moreover, the latter is more accepted for its conformity to discharge limits that are imposed by environmental regulatory authorities. For example, it meets the “class A” bio-solid product required by the United States Environmental Protection Agency (US EPA). 

Regarding advantages and disadvantages of mesophilic and thermophilic AD, Table 3 and Table 4 summarizes some of the points according to Kardos and and colleagues [24].

The effect of thermal pretreatment on the WAS is also investigated for its influence in biogas yield and dewatering of the sludge that is digested under the two temperatures of AD. In a study by Gavala et al., in 2003, thermophilic sludge AD was found to be effective on organic matter removal and methane yield under shorter digestion time. According to these investigators, the pre-treatment of sludge showed a positive effect on the rate of methane production by both mesophilic and thermophilic ADs. The authors, however, reported that pretreatment of sludge at 70 °C showed a more positive effect with the secondary sludge digested at a mesophilic temperature that would be due to the improved hydrolysis of suspended solids. The latter fact is also affirmed by other researchers, where thermal pretreatment is known to improve biogas yield by 21% and 31% [25]. 

According to Gavala et al., the improved stabilization and enhanced dewatering of sludge at a reasonable cost was mentioned as an advantage of thermal pretreatment; however, there are no detail provisions about it. These authors also underlined the effect of mixing primary sludge with secondary sludge to improve methane yield following the thermal pretreatment. Thus, there is an optimal mixing ratio corresponding to the methane yield in that regard.

Furthermore, the desirable effect of thermal pretreatment on methane production rate can be linked to the microbial diversity and abundance. To support this, the study by Khemkhao et al., using a Polymerase Chain Reaction-Based Denaturing Gradient Gel Electrophoresis analysis, suggested the suitability of thermophilic temperature to a number of hydrolytic, acidogenic, and acetogenic bacteria, which is suggested as compared to mesophilic temperature [26].

In a study by Yong Zhi Chi et al., WAS is digested in a semi continuous stirred tank reactor at mesophilic and thermophilic temperatures. This comparative process is operated under the following conditions: 4% total solid (TS), 30 days of hydraulic retention time (HRT), and OLR set at 1.67 kg-COD m^−3^·d^−1^ to both mesophilic and thermophilic ADs. As a result, they found out that the particulate organic matter reduction, and hence sludge volume reduction by the thermophilic reactor, is superior compared to the mesophilic AD. In addition to that, there was a slight increase of biogas yield by the thermophilic reactor; however, a statistically significant difference in mean methane content was observed in favor of the mesophilic AD [10]. 

As expected, the other recent full scale study by Cavinato et al. [12,27] showed that the biogas yield, and hence VS removal by the thermophilic digester, was found to be far better than the mesophilic counterpart; however, the methane content, expressed as percentage of the gas, showed no difference between the two temperatures. Uniquely, this study showed that there was no significant difference in process stability between the two thermal digesters through monitoring of the pH and alkalinity that is contrary to most related studies. In this same work, the increase in N-NH_4_^+^ was seen by the thermophilic AD; from 0.42 mg/L for the mesophilic to 0.69 mg/L for the thermophilic. The latter thus raises an issue regarding the need for further treatment [12,27].

A more focused experimental study to compare the mesophilic and thermophilic with regard to quality and quantity of biogas was conducted by Amani and co-workers in 2011. The study is conducted in batch-AD using WAS as substrate. In addition to biogas, the solid reduction, COD reduction, as well as VFA production and the pH were assessed. According to this group, the thermophilic AD improved the dewaterability of sludge, but no detail was given on that. However, both digesters showed accumulation of propionate, a factor in process stability. For obvious reasons because of the time-temperature combination effect, the time required to remove pathogens took longer in the mesophilic case. The seemingly preferable advantage in the case of mesophilic AD is the slightly higher amount of volumetric methane production over the thermophilic AD, though the biogas quality did not show a difference. This study is, however, a direct opposite of what was reported within a year by Yong Zhi Chi et al. However, the slightly higher volumetric methane production in the former is obtained at a longer HRT, due to the relatively stable synthrophic process as well as possible biomass autolysis. Nevertheless, cell lysis is obviously faster for thermophilic AD as the substrate concentration declines faster with time in the system and is also favoured by the relatively higher temperature effect. Therefore, the single relatively high volume of methane cannot surpass the faster biochemical process, low volume of digester required, and attainment of the US EPA’s “class A” solid advantages of thermophilic AD. Rather, the most limiting disadvantages according to Amani and colleagues is the energy required to heat digesters, highly polluted supernatants, and process instability in thermophilic AD, where the latter is still contrary to a study by Cavinto et al. [12]. In addition, the use of combined heat and power production results often brings a surplus of heat that can be reasonably used for increase of operational temperature.

With the advancement of WAS AD, a new technology called co-phasing that exchanges sludge between a spaced thermophilic and mesophilic digesters showed improved process stability and effluent quality as compared to the single stage mesophilic digesters. With the application of such technology, data on effluent quality showed increased NH_4_^+^-N (mg/L) by the thermophilic digesters, as was the case in the earlier studies. The issue of dewaterability is not addressed here except in mentioning poor effluent quality and low dewaterability for the thermophilic digesters. This study still contradicts the report by Amani et al., but here they used a different substrate [3,28]. Nevertheless, Amani and colleagues did not argue on why dewaterability is improved in the thermophilic case. Moreover, their argument on the effect of extracellular polymeric substance (EPS) contradicts their own claim on dewaterability. 

In a study by Suhartini and co-workers, thermophilic and mesophilic AD of sugar beet pulp was compared by varying the OLR over a long retention time. They found out that digestate dewaterability was better for the thermophilic AD. They also explained that the higher the OLR, the worse the dewaterability [29]. Thus, here it is worth mentioning that the dewaterability issue of sludge AD is of growing importance and the contradicting arguments in some reports raise the need for further research.

A seemingly ‘attractive’ earlier study by Moen et al. showed that the significant difference between the mesophilic and thermophilic AD of sludge underlay in the solid retention time (SRT) required the same digestion results. The difference is significant between the 6th and 20th days of the SRT. The statistically significant difference in VS destruction was observed at the 15th day of the SRT. Similar studies have been made reporting that over 90% of the biogas is produced within the first 14 days, and suggests this HRT as ideal for a batch AD as well. However, such a suggestion without specification of the digestion conditions and parameters is not acceptable [3,30]. The same study [30] also noted that the shorter the SRT, the more undigested VS remaining and hence higher sludge volume production. It is also observed that process instability, as determined from the pH and alkalinity measurements, is a function of the OLR. The higher the OLR, the higher the possibility of experiencing process instability. Contrarily, an earlier study by Zabranska and co-workers in 2000 noted that process stability is not a problem for thermophilic AD. Instead, they pointed to improved methane production rate and yield for the thermophilic primary sludge ADs. Nevertheless, the latter is made on a full scale AD of sludge and also with nutrient supplements [3]. 

Zabranska et al. also argued that the sudden increase in temperature created stress to the syntrophic conversion of acetate to methane that takes place at a faster rate than at lower temperatures. They also noted the relevance of thermophilic anaerobic systems in terms of lowering the demand for digester volume and sludge dewaterability, with no details about it. Regarding effluent quality, Nges and Liu [3] found out that thermophilic digesters discharge higher VFAs compared to the mesophilic ones. As outputs, the valeric, propionic and acetic acids were dominant, due to the faster hydrolysis followed by unbalanced consumption of the acids including those consumed by the methanogens. 

The other point that Zabranska and colleagues as well as other research advises on is that the change in substrate loading as well as temperature elevation has to be very gradual in order to give time for bacteria to adapt themselves with the change in process variables of the system. This issue has been evidenced by the decline in biogas yield in the early stage of temperature phasing, as authors mentioned. However, some argue on the rapid increase of temperature and gradual increase of loading as an alternative in this regard [9,30]. 

The typical reject water compositions are summarized by Ocansey (2005) in an unpublished Master’s Thesis as shown in Table 5. 

### 4.2. Sludge Dewaterability and Temperature of Digestion

The key aspect of sludge quality and costs for sludge disposal is its dewaterability. The typical dewaterability of the conventional WWTP sludge increases from activated sludge through raw to stabilized sludge as expressed in %TS; 15–25, 20–30, and 25–35, consecutively (Jenicek P.: unpublished survey of the Czech WWTPs). Thus, the stabilized sludge is the highest in dewaterability. In relation to this sludge stabilization through AD is an existing and energy producing, among its other benefits, alternative regarding sludge management. Such dewatering processes are also dependent on the temperature of digestion. 

As stated earlier, dewatering the sludge is useful for many reasons. These include minimizing the sludge volume, lowering the sludge transport cost, energy saving of matters associated with sludge drying and incineration, and improving sludge handling and sludge stability. The stabilizing advantage with the sludge dewatering is via moisture limiting, and hence limiting the moisture based microbial activity and the bounds of the resulting associated nuisances. Further sludge dewatering minimizes the amount of additives in cases of necessary bulking (composting, for example). The low dewaterability of sludge incurs a cost of sludge management, which takes a share of 25%–50% of the overall cost of wastewater treatment [32]. Thus, an efficient method of sludge treatment is very much needed. In a study by Zhou et al., dewaterability of sludge was significantly correlated to sludge EPS content [33].

Some articles published on this related aspect of sludge explain the poor settleability and dewaterability of anaerobic thermophilic sludge mainly due to high concentrations of dissolved COD. In addition, the thermophilic sludge has issues of excess ammonia in the reject water and related aspects but good sludge sanitary quality. The digestate is mainly a concern with regard to dewaterability and hydro-dynamicity that matter during pumping and transport of the sludge. Ge et al. mentioned in their study that thermophilic AD possesses better sludge quality in addition to improved gas yield [6]. 

In an advanced AD processes, sludge undergoes a chain of mainly thermal treatments to enhance the degree of stabilization. In a study by Braguglia et al., thermal pretreatment before AD of sludge worsened the dewaterability of the sludge, though it produced improved solid destruction and better biogas yield. In a related comparative study on mesophilic and thermophilic sludge AD with thickening as pretreatment and under specified operational conditions, the sludge dewaterability was worse for the thermophilic case despite its superiority in sludge reduction efficiency [10,34]. 

It is important to remind readers that, in those reported studies, a comprehensive and comparative study on the effect of temperature of digestion on anaerobic sludge quality, without sludge pretreatment, is not performed. Moreover, those limited studies on sludge dewaterability show some level of disparity and are also inconclusive. Thus, the aim of this review is to suggest the importance of a comprehensive and comparative study on anaerobic sludge quality, with respect to temperature of digestion, and come up with a clear picture of the difference in dewaterability, reject water characteristics and foaming effect that will serve as inputs to optimize sludge management in the WWTPs.

Sludge dewatering is not only limited to issues of volume minimization and subsequent liquor treatments. It is also important as sludge conditioning, which is a desired precondition for the subsequent other solids’ management, including odor removal, and in reducing the sludge putricibility. In an associated effort, various physicochemical approaches have been practiced to dewater sludge according to the US EPA. Those facts suggest that the thermal differential of AD would have an impact on the dewaterability of the sludge. Thus, if the effect of the temperature of sludge AD is found to be significant, it will have significant operational consequences. 

### 4.3. Sludge Pretreatment and Its Impact on Dewaterability

Unfortunately, most dewaterability related studies were not intended for comparison of mesophilic and thermophilic sludge ADs; rather, they are oriented to study the effect of physico-chemical and mechanical pretreatments on biodegradability and dewaterability as well as on sludge rheology. Among such studies, Liu et al. investigated the effect of sludge pretreatment with microwave-acid process, and they found improved sludge dewaterability with a capillary suction time (CST) [35]. 

In a related study, sludge floc disintegration using an ultrasound showed a decrease in dewaterability due to the increase of fine particles and bound water. As a result, the CST increased fifteen times. The lower the CST, the better is the dewaterability. Whereas, in this same study, the use of ozone as a pretreatment resulted in a slight increase in CST. Thus, the authors suggest that the optimization of both the degree of disintegration and the HRT are necessary to improve the dewaterability after disintegration [36]. It is also reported that low energy ultrasound treatment is generally used to improve sludge dewaterability [37]. 

EPS in the sludge is found to worsen the dewaterability of sludge after AD. With inoculation of selected microbes, Zhou et al. reduced the CST of sludge from 255.9 s to 25.45 s within 48 h in their work done in 2014. These EPSs are again impacted by the sludge composition, which is fed to digesters. Contrary to older papers from Zhou et al., the addition of glucose was found to increase the amount of EPSs produced, and it lowered the CST of sludge in an earlier study by Houghton and Stephenson. However, Houghton and Stephenson suggested an optimum level of EPS sought for better sludge dewaterability, with a value of 17.2 mg g^−1^ TSS [38]. The use of metal cations and chitosan to flocculate sludge after AD was found to improve the dewaterability of sludge, which could be part of a physicochemical post-treatment approach to sludge [33,39].

### 4.4. Sludge Management and Rheology

Since handling and management of sludge is a burdensome task for the existing and expanding WWTPs, it is highly demanded that cost effective dewatering alternatives are put in place as stated earlier in this document. The other most important sludge characteristics that need to be dealt also include physicochemical, sanitary and rheological aspects. These sludge properties affect the treatment of sludge, for example, by AD and the dewatering of sludge that are the principal processes in the WWTPs. Regarding rheological properties, anaerobically digested sludge is found to behave as a non-Newtonian fluid [40,41]. Therefore, manipulating the rheological property of sludge would affect the dewaterability of the sludge in one way or another. 

Viscosity, the major variable affecting the rheological property of sewage sludge, which is determined by measuring the sludge sheer stress, is significantly impacted by disintegration (up to 60%) of the sludge before digestion. In a related fact, digestion has a consequence on the rheological property of sludge as well. Sludge treatment, including its fermentation, also brings a change in its properties as a result of the changes in its complex composition. Rheological properties are very important in optimizing operations like sludge pumping and mixing. The mixing, transport or pumping and heat exchange system design during sludge management largely depends on the rheological property of the sludge. The rheological properties and quality of sludge liquor are affected by temperature [42]. Various researchers dealt with issues of dewaterability and other sludge physical characteristics mainly in relation to thermal treatment. 

A more rigorous study by Wang et al. showed that hydrothermal treatment of sludge at an elevated temperature, with a threshold temperature of 120–150 °C, gave a further reduction of 19%–47% of the moisture content compared to the one made at room temperature with a residence time of 30 min. In this research, the moisture content of the cake was in correlation with surface charge and hydrophobicity. The hydrothermal treatment also exhibited an energy advantage compared to the thermal drying of sludge [43]. 

The temperature of sludge treatment is also found to impact its hydrodynamic nature. This fact is studied by using the rheological property of the sludge following thermal treatment. However, other factors also matter in the fluidity of sludge, and the amount of the solid as one factor is worth mentioning here [1]. 

The essence of mixing in a biological reactor is all about bringing contact between microbes and substrate, which is highly desired for the optimum microbial activity, like in the case of the ADs. In sludge AD, getting that contact is a huge challenge, especially when it comes in to WAS as a substrate, which is mainly composed of biomass suspension where filamentous bacteria are one such obstacle. 

The non-Newtonian nature of untreated sludge is attributed to its composition expressed as electrostatic and gel-like interactions, and the presence of EPSs, which withhold water and contribute to sludge viscosity. Thus, the sludge thermal treatments result in the breakdown of macromolecules and larger particles, which improves dissolution of particles that again enhance the fluidity of sludge and increase the degree of dewatering due to the restructuring of the particles and release of the bound water in the sludge.

Due to the irreversible changes in composition of sludge at high temperatures mentioned above, it was proved that the higher the temperature, the more fluid the sludge. According to Urrea et al., if a temperature treatment of up to 200 °C is applied to sludge as a thermal treatment, the sludge starts behaving like a Newtonian fluid [44]. 

## 5. Conclusions

Though various aspects of sludge AD have been studied under different conditions, an integrative and holistic approach must be applied with emphasis on operational temperature of digestion. Given the faster rate of sludge digestion and shorter SRT in the reactor, the use of thermophilic AD is often preferred over mesophilic AD. Thermophilic digestion is also capable of meeting the US EPA’s “class A” solid requirements in digested sludge. Nevertheless, the operational conditions during the use of the CSTRs are restrictive compared to the mesophilic AD. 

Though process stability issues favor mesophilic systems, in many cases, it is of less concern with regard to waste activated sludge AD. However, the digestate quality and reject water properties may clearly limit the thermophilic mode compared to mesophilic AD. Many studies in these regards are inconclusive and somehow contradictory. 

Furthermore, most studies done on the AD of sludge are not comprehensive enough in relation to the complete data about operational factors, and no clear decisive criterion is put in place to prefer one system over the other with respect to the two temperature categories—thermophilic and mesophilic. Thus, a comprehensive approach should encompass most, if not all, of the relevant process aspects or variables. Therefore, there is the need for a study on the optimization of the most important operational aspects such as that of biogas yield, sludge dewaterability, and the reject water quality for an informed choice between the two AD temperature systems.

## Figures and Tables

**Table 1:** The composition of wastewater sludge with respect to the treatment applied to it.

Content	Unit	Class				
		A	B1	B2	C	D
Dry matter	g/L	12	9	7	10	30
Volatile matter	%DM	65	67	77	72	50
pH	Scale	6	7	7	6.5	7
C	%VM	51.5	52.5	53	51	49
H	%VM	7	6	6.7	7.4	7.7
O	%VM	35.5	33	33	33	35
N	%VM	4.5	7.5	6.3	7.1	6.2
S	%VM	1.5	1	1	1.5	2.1
C/N	Unit less	11.4	7	8.7	7.2	7.9
P	%DM	2	2	2	2	2
Cl	%DM	0.8	0.8	0.8	0.8	0.8
K	%DM	0.3	0.3	0.3	0.3	0.3
Al	%DM	0.2	0.2	0.2	0.2	0.2
Ca	%DM	10	10	10	10	10
Fe	%DM	2	2	2	2	2
Mg	%DM	0.6	0.6	0.6	0.6	0.6
Fat	%DM	18	8	10	14	10
Protein	%DM	24	36	34	30	18
Fibers	%DM	16	7	10	13	10
Calorific value	kWh/tDM	4200	4100	4800	4600	3000

(Source: the European Commission (2001) after OTV, 1997) [13].

**Table 2:** Typical process stability parameters and control values.

Processcondition	Parameter	Value	Author
Propionateoxidation	Hydrogen Partial pressure	10^−4^ to 10^−6^ atmosphere	McCarthy & Smith, 1986
Ethanol oxidation	Hydrogen Partial pressure	10^−1^ to 10^−6^ atmosphere	McCarthy & Smith, 1986
Total processinhibition	Free ammonia	10 g-N/L	Apples et al., 2008
Inhibition of 50% methanogens	Free ammonia	560–568 mg NH_3_-N/L	Apples et al., 2008
Significantinhibitiontomethanogens	Propionicacidconcentration	900 mg/L	Whittle et al., 2014

**Table 3:** Corresponding advantages of mesophilic and thermophilic digestion systems. Source: [24].

Mesophilic System	Thermophilic System
During the biogas production organic material is stabilizing, fermented sludge can be applied as dung	Increased gas output due to the faster reaction; higher methane gas content and reduces hydrogen sulfide content in the biogas
Sludge’s quantity reducing	Staying-duration shorter
Sludge’s fertilization ability reducing	Smaller reactor volume demand
Sludge’s water down take capacity getting better	More pathogen destruction
	Sludge’s dehydratation getting better
	Reduced foam formation in the reactor

**Table 4:** Respective disadvantages of mesophilic and thermophilic digestion systems. Source: [24].

Mesophilic System (Related to the Unstabilised Sludge)	Thermophilic System (Related to the Mesophilic System)
Due to the longer staying duration–larger reactor volume demand, higher investment’s costs	Higher heater energy demand
Sludge water’s quality getting worse	Sludge water’s quality getting worse
Fermentation blocking influence of heavy metals	Sensitivity to the sudden temperature fluctuation, more precise temperature regulation demand
	sensitivity to the toxic heavy metals

**Table 5:** Typical composition of reject water.

Reject Water Parameter	Unit	Typical Range
N_Kj_	mg/L	690–1700
TAN	mg/L	600–1513
Total Phosphorus	mg/L	Trace-130
TSS	mg/L	<800
COD	mg/L	700–1400
Temperature	°C	25–40
pH	Scale	7–13
Alkalinity_4.5_	mmol/L	53–150

Source: [31], N_Kj_—Kjeldahl nitrogen, (sum of TAN and organic nitrogen), TAN—total ammonia nitrogen.

## References

[1] Jiang J., Wu J., Poncin S., Li H.Z. (2014). Rheological characteristics of highly concentrated anaerobic digested sludge. Biochem. Eng. J..

[2] Appels L., Baeyens J., Degrève J., Dewil R. (2008). Principles and potential of the anaerobic digestion of waste-activated sludge. Prog. Energy Combust. Sci..

[3] Nges I.A., Liu J. (2010). Effects of solid retention time on anaerobic digestion of dewatered-sewage sludge in mesophilic and thermophilic conditions. Renew. Energy.

[4] Verstraete W., Vlaeminck S.E. (2011). ZeroWasteWater: Short-cycling of wastewater resources for sustainable cities of the future. Int. J. Sustain. Dev. World Ecol..

[5] Zábranská J., Dohányos M., Jenícek P., Kutil J. (2000). Thermophilic process and enhancement of excess activated sludge degradability—Two ways of intensification of sludge treatment in the Prague central wastewater treatment plant. Water Sci. Technol..

[6] Ge H., Jensen P.D., Batstone D.J. (2011). Relative kinetics of anaerobic digestion under thermophilic and mesophilic conditions. Water Sci. Technol..

[7] Gavala H.N., Yenal U., Skiadas I.V., Westermann P., Ahring B.K. (2003). Mesophilic and thermophilic anaerobic digestion of primary and secondary sludge. Effect of pre-treatment at elevated temperature. Water Res..

[8] McCarty P.L., Smith D.P. (1986). Anaerobic wastewater treatment. Environ. Sci. Technol..

[9] De la Rubia M.A., Riau V., Raposo F., Borja R. (2013). Thermophilic anaerobic digestion of sewage sludge: Focus on the influence of the start-up. A review. Crit. Rev. Biotechnol..

[10] Chi Y.Z., Li Y.Y., Ji M., Qiang H., Deng H.W., Wu Y.P. (2010). Mesophilic and Thermophilic Digestion of Thickened Waste Activated Sludge: A Comparative Study. Adv. Mater. Res..

[11] Amani T., Sreekrishnan T.R. (2011). Experimental Study on Key Dissimilarities between Mesophilic and Thermophilic Anaerobic Digestion of Waste Activated Sludge. Int. J. Environ. Res..

[12] Cavinato C.B.D., Pavan P., Fatone F., Cecchi F. (2013). Mesophilic and thermophilic anaerobic co-digestion of waste activated sludge and source sorted biowaste in pilot- and full-scale reactors. Renew. Energy.

[13] Bitton G. (2005). Anaerobic Digestion of Wastewater and Biosolids. Wastewater Microbiology.

[14] Ho D.P., Jensen P.D., Batstone D.J. (2013). Methanosarcinaceae and acetate-oxidizing pathways dominate in high-rate thermophilic anaerobic digestion of waste-activated sludge. Appl. Environ. Microbiol..

[15] Weedermann M., Seo G., Wolkowicz G.S.K. (2013). Mathematical model of anaerobic digestion in a chemostat: Effects of syntrophy and inhibition. J. Biol. Dyn..

[16] Lee M.J., Zinder S.H. (1988). Hydrogen Partial Pressures in a Thermophilic Acetate-Oxidizing Methanogenic Coculture. Appl. Environ. Microbiol..

[17] Kaspar H., Wuhrmann K. (1977). Product inhibition in sludge digestion. Microb. Ecol..

[18] Sung S., Liu T. (2003). Ammonia inhibition on thermophilic anaerobic digestion. Chemosphere.

[19] Rajagopal R., Massé D.I., Singh G. (2013). A critical review on inhibition of anaerobic digestion process by excess ammonia. Bioresour. Technol..

[20] Franke-Whittle I.H., Walter A., Ebner C., Insam H. (2014). Investigation into the effect of high concentrations of volatile fatty acids in anaerobic digestion on methanogenic communities. Waste Manag..

[21] Kim M., Ahn Y.H., Speece R.E. (2002). Comparative process stability and efficiency of anaerobic digestion; mesophilic *vs.* thermophilic. Water Res..

[22] Li Q., Qiao W., Wang X., Takayanagi K., Shofie M., Li Y.-Y. (2015). Kinetic characterization of thermophilic and mesophilic anaerobic digestion for coffee grounds and waste activated sludge. Waste Manag..

[23] Kardos L., Juhász Á., Palkó G., Oláh J., Barkács K., Záray G. (2011). Comparing of thermophilic and mesophilic anaerobic fermented sewage sludge based on chemical and biochemical tests. Appl. Ecol. Environ. Res..

[24] Ruffino B., Campo G., Genon G., Lorenzi E., Novarino D., Scibilia G., Zanetti M. (2015). Improvement of anaerobic digestion of sewage sludge in a wastewater treatment plant by means of mechanical and thermal pre-treatments: Performance, energy and economical assessment. Bioresour. Technol..

[25] Khemkhao M., Nuntakumjorn B., Techkarnjanaruk S., Phalakornkule C. (2012). Comparative mesophilic and thermophilic anaerobic digestion of palm oil mill effluent using upflow anaerobic sludge blanket. Water Environ. Res..

[26] Song Y.C., Kwon S.J., Woo J.H. (2004). Mesophilic and thermophilic temperature co-phase anaerobic digestion compared with single-stage mesophilic- and thermophilic digestion of sewage sludge. Water Res..

[27] Suhartini S., Heaven S., Banks C.J. (2014). Comparison of mesophilic and thermophilic anaerobic digestion of sugar beet pulp: Performance, dewaterability and foam control. Bioresour. Technol..

[28] Moen G., Stensel H.D., Lepistö R., Ferguson J.F. (2003). Effect of Solids Retention Time on the Performance of Thermophilic and Mesophilic Digestion of Combined Municipal Wastewater Sludges. Water Environ. Res..

[29] Ocansey F.N. (2005). New Trends In Treatment of Reject Water from Dewatering of Sludge. Master’s Thesis.

[30] Zhou J., Zheng G., Zhang X., Zhou L. (2014). Influences of extracellular polymeric substances on the dewaterability of sewage sludge during bioleaching. PLoS ONE.

[31] Zhou J., Mavinic D.S., Kelly H.G., Ramey W.D. (2002). Effects of temperatures and extracellular proteins on dewaterability of thermophilically digested biosolids. J. Environ. Eng. Sci..

[32] Braguglia C.M., Gianico A., Gallipoli A., Mininni G. (2015). The impact of sludge pre-treatments on mesophilic and thermophilic anaerobic digestion efficiency: Role of the organic load. Chem. Eng. J..

[33] Liu J.B., Ni X.T., Wei Y.S., Tong J., Wang Y.W. (2014). Enhancement for anaerobic digestion of sewage sludge pretreated by microwave and its combined processes. Environ. Sci..

[34] Braguglia C.M., Gianico A., Mininni G. (2009). Effect of ultrasound on particle surface charge andfilterability during sludge anaerobic digestion. Water Sci. Technol..

[35] Braguglia C.M., Gianico A., Mininni G. (2012). Comparison between ozone and ultrasound disintegration on sludge anaerobic digestion. J. Environ. Manag..

[36] Houghton J.I., Stephenson T. (2002). Effect of influent organic content on digested sludge extracellular polymer content and dewaterability. Water Res..

[37] Lau S.W., Chong S.H., Ang H.M., Sen T.K., Chua H.B., Pogaku R., Bono A., Chu C. (2013). Dewaterability of Anaerobic Digested Sludge with Cations and Chitosan as Dual Conditioners. Developments in Sustainable Chemical and Bioprocess Technology.

[38] Baudez J.C.M.F., Eshtiaghi N., Slatter P. (2011). The rheological behaviour of anaerobic digested sludge. Water Res..

[39] Aranowski R., Hupka J., Jungnickel C. (2010). Changes in Rheological Properties during Anaerobic Digestion of Activated Sludge. Physicochem. Probl. Miner. Process..

[40] Farno E., Baudez J.C., Parthasarathy R., Eshtiaghi N. (2014). Rheological characterisation of thermally-treated anaerobic digested sludge: Impact of temperature and thermal history. Water Res..

[41] Feng G., Tan W., Zhong N., Liu L. (2014). Effects of thermal treatment on physical and expression dewatering characteristics of municipal sludge. Chem. Eng. J..

[42] Wang L., Zhang L., Li A. (2014). Hydrothermal treatment coupled with mechanical expression at increased temperature for excess sludge dewatering: Influence of operating conditions and the process energetics. Water Res..

[43] Urrea J.L., Collado S., Laca A., Díaz M. (2015). Rheological behaviour of activated sludge treated by thermal hydrolysis. J. Water Process Eng..

[44] Disposal and Recycling Routes for Sewage Sludge, part-3 Scientific and Technical Report. http://ec.europa.eu/environment/archives/waste/sludge/pdf/sludge_disposal3.pdf..

